# Is severe maternal morbidity a risk factor for postpartum hospitalization with mental health or substance use disorder diagnoses? Findings from a retrospective cohort study in Maryland: 2016–2019

**DOI:** 10.21203/rs.3.rs-4655614/v1

**Published:** 2024-07-23

**Authors:** Carrie L WOLFSON, Jessica Tsipe ANGELSON, Andreea A CREANGA

**Affiliations:** Johns Hopkins Bloomberg School of Public Health; Johns Hopkins Bloomberg School of Public Health; Johns Hopkins Bloomberg School of Public Health

**Keywords:** Postpartum depression, substance use disorder, maternal health, postpartum hospitalization

## Abstract

**BACKGROUND:**

Perinatal mental health conditions and substance use are leading causes, often co-occurring, of pregnancy-related and pregnancy-associated deaths in the United States. This study compares odds of hospitalization with a mental health condition or substance use disorder or both during the first year postpartum between patients with and without severe maternal morbidity (SMM) during delivery hospitalization.

**Methods:**

Data are from the Maryland’s State Inpatient Database and include patients with a delivery hospitalization during 2016–2018 (n = 197,749). We compare rate of hospitalization with a mental health condition or substance use disorder or both at 42 days and 42 days to 1 year postpartum by occurrence of SMM during the delivery hospitalization. We use multivariable logistic regression to derive the odds of hospitalization with each outcome for patients by SMM status, adjusted for patient sociodemographic characteristics, presence of mental health condition or substance use disorder diagnoses during the delivery hospitalization, and delivery outcome. SMM, mental health conditions, and substance use disorders are identified using ICD-10 diagnosis and procedure codes.

**RESULTS:**

Overall, 5,793 patients (2.9%) who delivered during 2016–2018 experienced hospitalization in the year following delivery. Among these patients, 24.3% (n = 1,410) had a mental health condition diagnosis, 10.6% (n = 619) had a substance use disorder diagnosis, and 9.8% (n = 570) had co-occurring mental health condition and substance use disorder diagnoses. Patients with SMM had 3.7 times the odds (95% CI 2.7, 5.2) of hospitalization with a mental health condition diagnosis, 2.7 times the odds (95% CI 1.6, 4.4) of a hospitalization with substance use disorder diagnosis, and 3.0 times the odds (95% CI 1.8, 4.8) of hospitalization with co-occurring mental health condition and substance use disorder diagnoses during the first-year postpartum adjusting for covariates.

**CONCLUSION:**

Patients who experience SMM during their delivery hospitalization had higher odds of hospitalization with a mental health condition, substance use disorder, and co-occurring mental health condition and substance use disorder in the one-year postpartum period. Treatment and support resources for mental health and substance use providers --including enhanced screening and warm handoffs -- should be made available to patients with SMM upon discharge after delivery, and evidence-based interventions to improve mental health and reduce substance use should be prioritized in these patients.

## BACKROUND

The prevalence of perinatal mental health conditions, including depression, anxiety, bipolar disorder, and suicidality, has doubled in the past two decades in the United States, affecting more than 20% of pregnant and postpartum people annually (1–4). Perinatal substance use and substance use disorders have also risen dramatically, with 6.5 cases of opioid use disorder reported per 1,000 delivery hospitalizations in 2016 (5, 6). In fact, mental health conditions and substance use disorders are leading causes of pregnancy-related and pregnancy-associated death in the United States (7, 8), and have also been associated with poor maternal, neonatal, and infant outcomes (9) and increased delivery hospitalization costs (10, 11). Notably, presence of a mental health condition diagnosis during the delivery hospitalization has been shown to be associated with an increased likelihood of severe maternal morbidity (SMM) (3, 10, 12), the rate of which also increased by 22.5% between 2008 and 2021 in the US and is known to have long-term consequences on women’s health (13, 14). Less is known about the relationship between SMM and postpartum mental health, although recent studies indicate an increased risk of such conditions following an SMM event (15, 16). These findings are consistent with qualitative evidence in which patients describe their SMM events as having long-term negative psychological impacts (17–19). Despite the recognized co-occurrence of mental health conditions and substance use disorders (20, 21), these conditions are not usually examined in tandem among peripartum individuals.

Data from all hospital admissions within one year postpartum in Maryland offer the opportunity of exploring the association between SMM and postpartum mental health and substance use, both separately and as co-occurring morbidities. The objective of this study is to examine rates of hospitalization with a mental health condition, or substance use disorder, or both during the first year postpartum among patients with and without SMM during delivery hospitalization in Maryland over a 4-year period before the start of the COVID-19 pandemic.

## MATERIALS AND METHODS

We conducted a retrospective cohort study using 2016–2019 Maryland State Inpatient Database (SID) maintained by the Agency for Healthcare Research and Quality for the Healthcare Cost and Utilization Project (HCUP). Delivery discharge records were identified using the International Classification of Diseases 10th Revision, Clinical Modification (ICD-10-CM) diagnosis codes and procedure codes among patients identified as female and of reproductive age (15–49 years). Postpartum hospitalizations were identified using a unique patient identifier which is consistent across admissions and facilities over time in the Maryland SID.

The 3 primary outcomes for the analysis were postpartum hospital admission with a mental health condition, a substance use disorder, and both. Outcome assessment was based on the presence of at least one ICD-10-CM diagnosis code for a mental health condition or substance use disorder among the first five ICD-10-CM diagnosis codes listed for each patient (a full list of ICD-10-CM diagnosis codes used to identify outcomes are included in Appendix 1); of note, SID allows up to 30 diagnosis and procedure ICD codes to be listed for each hospital discharge. Within the sample of patients with at least one delivery during 2016–2018, 82.2% of records included at least 5 diagnosis codes, 70.0% had 6 or more diagnosis codes. For deriving the co-occurring disorders outcome, we included individuals with a mental health condition or a substance use disorder diagnosis among the first 5 diagnoses listed who also had a diagnosis code for the other type of disorder listed for the same hospital discharge. Outcomes were identified from the day after discharge from the delivery hospitalization through 365 days following delivery. To examine outcomes one year postpartum, the sample was restricted to patients with deliveries occurring during 2016–2018 to allow for sufficient follow up time in 2019 data.

Severe maternal morbidity (SMM) was defined using the Center for Disease Control and Prevention’s SMM ICD-10-CM algorithm based on ICD-10-CM diagnoses and procedures noted during the delivery hospitalization (14). In line with the most recent literature, patients with only blood transfusion ICD-10-CM codes were not deemed as having experienced SMM due to poor positive predictive value of SMM using ICD-10-CM codes for this specific indicator (22). Patient sociodemographic information available and used in this analysis includes age, race/ethnicity (categorized as non-Hispanic Black, non-Hispanic White, Hispanic, and other/multiple races using HCUP’s standard variables for race and ethnicity), income quartile for the patient’s zip code, delivery hospitalization payer (private, public, self-pay/none), and location of residence (urban or rural). We also identified the presence of a maternal mental health condition, or a substance use disorder noted during delivery hospitalization, as well as the delivery outcome (stillbirth or live birth).

These characteristics were compared between patients with and without SMM using Pearson chi-square analyses. Rates of hospitalization during the early (within 42 days) and late (42–365 days) postpartum periods were calculated for patients with and without SMM and for each outcome of interest (hospitalization with a mental health condition, a substance use disorder, and co-occurring mental health condition and substance use disorder). Unadjusted and multivariable logistical regression models were fitted to assess the relationship between SMM during delivery hospitalization and postpartum hospitalization with one of the 3 outcomes of interest during the first postpartum year. Adjusted models controlled for patient age, race and ethnicity, median household income for zip code, insurance type, urban/rural residence, presence of a mental health condition or a substance use disorder during delivery, and birth outcome, as well as clustering at the patient level using cluster subcommands to account for patients with multiple deliveries during the period of analysis (n = 26,930; 15.9% of patients with deliveries between 2016 and 2018 in the sample). Analyses were conducted using Stata 15.1 software (College Station, TX).

## RESULTS

A total of 197,749 delivery hospitalizations were identified in Maryland between 2016 and 2018; 0.8% (n = 1,513) involved SMM. Compared to patients without SMM, a higher proportion of patients with SMM were 35–39 years of age (21.6% vs. 17.1%, respectively) and 40 years of age or older (8.3% vs. 3.9%, respectively) (Table 1). Patients with SMM were also in higher proportion non-Hispanic Black (46.1% vs. 30.5%, respectively), with incomes in lowest income quartile for ZIP code (33.4% vs. 27.8%, respectively), and covered by public insurance (49.1% vs. 42.4%, respectively) than those without SMM. Also, a higher proportion of patients with than without SMM had a mental health condition (17.1% vs. 9.4%, respectively) or substance use disorder (6.9% vs. 3.6%, respectively) diagnosis documented during the delivery hospitalization. Additionally, the outcome of deliveries complicated by SMM were in higher proportion stillbirth in patients with SMM compared to patients without SMM (6.5% vs. 0.7%, respectively).

**Figure 1:**
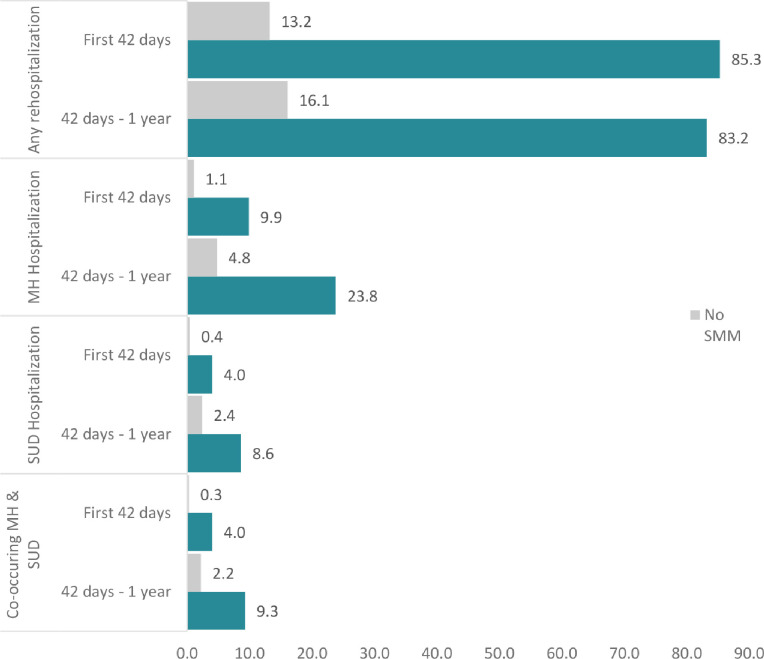
Postpartum Hospitalization Rates Overall and among Patients with a Mental Health Condition or Substance Use Disorder Diagnoses by Severe Maternal Morbidity Status During Delivery Hospitalization Note: SMM, Severe Maternal Morbidity; MH, mental health; SUD, substance use disorder. Postpartum hospitalization rate is number of hospitalizations following delivery hospitalization discharge per 1,000 deliveries. Source: Healthcare Cost and Utilization Project (HCUP), Maryland Inpatient Database, 2016–2019. N=197,749 deliveries in 2016–2018.

Among patients with deliveries, 2.9% (n = 5,793) experienced hospitalization in the year following delivery. The rate of postpartum hospitalization was higher across all outcomes and time periods for patients with SMM compared to those without SMM (Fig. 1). The relative risk of early postpartum hospitalization for patients with SMM was 6.5 for any hospitalization (95% CI = 5.4–7.6), 8.8 for a hospitalization with a mental health condition diagnosis (95% CI = 5.2–14.7), 9.9 for a substance use disorder diagnosis (95% CI = 4.3, 22.6), and 13.6 for both (95% CI = 5.9–31.6) compared to patients without SMM. In the late postpartum period, the relative risk of postpartum hospitalization between patients with and without SMM for any cause was 5.2 (95% CI = 4.4, 6.1). The relative risk was 4.9 (95% CI = 3.5, 6.9), 3.6 (95% CI = 2.1, 6.2), and 4.1 (95% CI = 2.4, 7.0) for hospitalizations with a mental health condition, substance use disorder, and both, respectively. The rate of hospitalization for any reason was higher in the early postpartum than in the late postpartum period for patients with SMM (85.3 per vs. 83.2 1,000 deliveries, respectively). However, among all patients (with and without SMM) the rate of postpartum hospitalization with a mental health condition or substance use disorder was higher in the late postpartum period.

Compared with patient without SMM, those with SMM had significantly higher odds of postpartum hospitalization with a mental health condition diagnosis (OR = 5.7; 95% CI 4.2, 7.6), a substance use disorder (OR = 4.4; 95% CI 2.8, 7.1) and co-occurring mental health conditions and substance use disorder diagnoses (OR = 4.9, 95% CI 3.1, 7.9) (Table 2). Odds were attenuated by 34.2–40.2% after adjustment for the sociodemographic and delivery characteristics examined.

Several sociodemographic and delivery characteristics were associated with the odds of postpartum hospitalization with a mental health condition or substance use disorder or both types of diagnoses. Compared to non-Hispanic White patients, patients who were Hispanic had a lower odds of postpartum hospitalization with a mental health condition (AOR = 0.5, 95% CI 0.4, 0.6), substance use disorder (AOR = 0.2, 95% CI 0.1, 0.3), or co-occurring mental health condition and substance use disorder (AOR = 0.2, 95% CI 0.1, 0.3) compared to patients who were non-Hispanic White. Though patients who were non-Hispanic Black had approximately 20% higher odds of postpartum hospitalization with a mental health condition compared to non-Hispanic White patients (in unadjusted models only), they had approximately 30% lower odds of postpartum hospitalization with a substance use disorder or co-occurring mental health condition and substance use disorder compared to non-Hispanic White patients.

By and large, the higher the patient income, the lower the odds of postpartum hospitalization with mental health condition or substance use disorder diagnoses. Compared to patients with private insurance, those with public insurance or no insurance had 1.9 times higher odds of postpartum hospitalization with a mental health condition diagnosis and over 5-times higher odds of postpartum hospitalization with substance use alone or both substance use and mental health condition diagnoses. Importantly, odds of postpartum hospitalization with a mental health condition diagnosis were significantly higher for patients with a mental health condition (AOR = 6.6; 95% CI 5.8, 7.5) or substance use disorder (AOR = 2.1; 95% CI 1.7, 2.5) diagnosis during the delivery hospitalization. Patients with a stillbirth had 2.2 times higher odds for postpartum hospitalization with a mental health condition diagnosis compared to patients with a live born infant.

## DISCUSSION

In this retrospective cohort study of deliveries in Maryland between 2016–2018, patients who experienced SMM during their delivery hospitalization had 3.7, 2.8 and 3 times the odds of postpartum hospitalization with a mental health condition diagnosis, substance use disorder diagnosis and both, respectively, compared to patients who did not experience SMM during delivery. Rates of postpartum hospitalization with such diagnoses were significantly higher among patients with SMM compared to those without SMM during the early and late postpartum periods. Our findings confirm prior research documenting higher rates of postpartum hospitalization among postpartum patients who experience SMM compared to those who did not experience SMM (23), and are supported by clinical plausibility with respect to the associations between SMM and presence of mental health condition and substance use disorder diagnoses. Research has shown that traumatic birth outcomes increase the risk of mental health conditions, particularly posttraumatic stress disorder, during the postpartum period (24–26). Moreover, a study in Sweden found a positive association between SMM at delivery and postpartum treatment for psychiatric disorders (27). Experience of adverse or unexpected outcomes such as preeclampsia, preterm birth, or cesarean delivery have been identified as risk factors for postpartum mental health conditions (28–30). Similarly, the postpartum period is recognized as a particularly vulnerable time for individuals with substance use disorders at risk of relapse, which may be further compounded by the experience of birth trauma or adverse pregnancy outcomes (31). Fear for personal safety or safety of the neonate and negative perceptions of birth, multiple interventions during labor and birth, and anesthesia complications are all associated with postpartum mental health conditions and can be unique vulnerabilities for those with histories of substance use disorders (31, 32). SMM frequently includes multiple interventions as well as anesthesia and is often characterized as a traumatic event by those who experience them, which can explain the relationships we observed in our study.

Efforts to reduce preventable SMM and its effects should include recognition and management of mental health and substance use in pregnancy and postpartum, and particularly the late postpartum period. In our study, postpartum hospitalization rates for patients with mental health and substance use disorders were higher during the late postpartum period compared to the early postpartum period. Similarly, studies of pregnancy-associated mortality have identified higher rates of deaths due to overdose and suicide in the late postpartum period (33, 34). Together, these suggest the late postpartum period is a particularly vulnerable time for these behavioral health conditions.

Patients with SMM often receive inadequate information about their morbidity (35). Recommendations following an SMM event include offering patients a debriefing by their clinician before hospital discharge, social support, referrals and warm hand-off to mental health and substance use providers and services, including specialized treatment (35). Such interventions have been found to be moderately effective in reducing mental health symptoms among patients with traumatic births (36, 37). The US Preventive Services Task Force recommends that clinicians provide or refer postpartum persons at increased risk for perinatal depression to counseling interventions due to sufficient evidence of their effectiveness (38). Increased risk includes those with a history of depression, current depressive symptoms, socioeconomic risk factors, or a history of significant negative life events. Findings from this analysis suggest that experience of SMM should also be considered as a risk factor.

Our findings demonstrate no significant difference in the in adjusted odds of postpartum hospitalization with a mental health condition between non-Hispanic Black and White patients who experience SMM, but lower odds of postpartum hospitalization for substance use disorders and both mental health conditions and substance use disorders. Additionally,, odds of hospitalization with a mental health condition or substance use disorder were significantly lower among Hispanic patients and other racial/ethnic minority groups compared to white patients. More research is needed to determine whether these differences are due to true lower rates of mental health conditions and substance use disorders or differences in rates of diagnosis and recognition.

Our study has limitations. While there are indications that SMM precipitates postpartum mental health conditions and substance use, these relationships may be bidirectional, such that a history of mental health conditions or substance use predisposes pregnant individuals to SMM, which in turn exacerbates the risk of the same in the postpartum period (16). Clearly establishing the directionality and causality of this relationship will require further, more robust longitudinal studies. In addition, examining the relationship of SMM with specific types of mental health conditions as well as co-occurring mental health conditions and substance use should be the focus of research when a larger sample size than ours is available. We used the first five diagnosis codes for each patient to ascertain our outcomes of interest – this cut-off point was used to examine postpartum hospitalizations where mental health conditions and substance use disorders represented important reason(s) for the hospitalizations. However, we may have missed patients who were admitted to the hospital for these conditions but, for which, only direct pregnancy complications were noted with the first five conditions codes. We may have also missed patients who do not disclose their mental health condition or symptoms, or their substance use to a healthcare provider, or those who are less likely to be screened for such conditions including birthing people of color and those with low incomes (39). The data are limited to conditions identified during hospitalizations, whereas many of these conditions do not result in hospitalization and may be identified in outpatient settings and treated through medication, therefore we are unable to adjust for prenatal depression and substance use, unless identified during the delivery hospitalization; moreover, only SMM during delivery hospitalization is examined, thus excluding SMM events that occur in antepartum and postpartum hospitalizations. In addition, residual confounding may be present due to the exclusion of other chronic health conditions and the limited sociodemographic variables available through hospital records. Furthermore, reliance on administrative data, which are primarily collected for billing purposes, has other limitations because all health conditions present during a hospital admission may not be consistently and accurately reported and the algorithm used to identify SMM based on this data is not well suited to identify all cases of SMM events involving hemorrhage (22).

However, the study has some important strengths. It uses a large, statewide database analyzed longitudinally over several years before the start of the COVID-19 pandemic – this was only possible given the inclusion of a unique patient identifier in the Maryland SID. The use of ICD-10 codes for outcome ascertainment has the strength of consistency in identifying cases over the study period and offers some reassurance that they represent true cases of patients with mental health conditions or substance use disorders.

## CONCLUSION

Patients who experience SMM have a higher odds of hospitalization with a mental health condition or substance use disorder or both in the postpartum period. Treatment and support resources for mental health and substance use providers --including enhanced screening and warm handoffs -- should be made available to patients with SMM upon discharge after delivery, and evidence-based interventions to improve mental health and reduce substance use should be prioritized in these patients.

## Figures and Tables

**Table 1:** Characteristics of Patients with and without Severe Maternal Morbidity During Delivery Hospitalization

	No SMM (n = 196,236)		SMM (n = 1,513)		P-value for difference
	N	%	N	%	
Age (years)					<0.000
<25	38,774	19.8	289	19.1	
25–29	54,167	27.6	334	22.1	
30–34	62,151	31.7	439	29.0	
35–39	33,565	17.1	326	21.6	
40+	7,579	3.9	125	8.3	
Race & Ethnicity					<0.000
NH Black	59,838	30.5	697	46.1	
NH White	84,723	43.2	462	30.5	
Hispanic	29,683	15.1	195	12.9	
Other/multiple races	21,992	11.2	159	10.5	
Primary Language					0.453
English	118,996	60.6	872	57.6	
Non-English	12,473	6.4	99	6.5	
Missing/unknown	64,767	33.0	542	35.8	
Median household income^1^					<0.000
1st quartile	54,549	27.8	505	33.4	
2nd quartile	53,346	27.2	426	28.2	
3rd quartile	48,437	24.7	328	21.7	
4th quartile	39,374	20.1	252	16.7	
Missing/unknown	530	0.3	*	< .1	
Urban residence					0.217
Urban	191,252	97.5	1,483	98.0	
Rural	4,860	2.5	30	2.0	
Missing/unknown	124	0.1	0	0	
Primary insurance					< 0.000
Private	104,314	53.2	694	45.9	
Public	83.163	42.4	743	49.1	
Self-pay/none	8,675	4.4	75	5.0	
Missing/unknown	84	<0.1	*	<0.1	
Comorbidities^2^					
Mental health condition	18,431	9.4	258	17.1	<0.000
Substance use	7,107	3.6	104	6.9	<0.000
Stillbirth	1,346	0.7	99	6.5	<0.000

Note: SMM, Severe Maternal Morbidity; NH, non-Hispanic.

1 Median household zip code calculated based on zip code of patient’s residence.

2 Indicated during delivery hospitalization.

P-value based on chi-square analysis of difference in distribution between patients with and without SMM during the delivery hospitalization.

* Restricted due to small cell size.

Source: Healthcare Cost and Utilization project (HCUP) Maryland Inpatient Database, 2016–2018; N = 197,749 deliveries.

**Table 2:** Odds of Postpartum Hospitalization for Patients with a Mental Health Condition or Substance Use Disorder Diagnoses by Severe Maternal Morbidity Status

	Hospitalization with Mental Health Condition				Hospitalization with Substance Use Disorder				Hospitalization with Co-Occurring MH & SUD			
	Unadjusted		Adjusted		Unadjusted		Adjusted		Unadjusted		Adjusted	
	OR	95% CI	OR	95% CI	OR	95% CI	OR	95% CI	OR	95% CI	OR	95% CI
SMM during delivery (no SMM = Ref.)	5.67	4.21,7.64	3.73	2.69,5.16	4.44	2.77,7.1	2.67	1.62,4.4	4.93	3.08,7.9	2.95	1.81,4.8
Age (years)												
<25	Ref.		Ref.		Ref.		Ref.		Ref.		Ref.	
25–29	0.61	0.53,0.72	0.71	0.61,0.83	0.87	0.7,1.07	1.05	0.84,1.3	0.79	0.63,0.98	0.97	0.77,1.22
30–34	0.49	0.42,0.58	0.72	0.61,0.85	0.48	0.38,0.61	0.86	0.67,1.11	0.41	0.31,0.52	0.74	0.57,0.97
35–39	0.48	0.4,0.58	0.73	0.6,0.89	0.43	0.32,0.59	0.90	0.66,1.24	0.43	0.32,0.58	0.91	0.66,1.25
40+	0.38	0.26,0.56	0.61	0.41,0.9	0.48	0.28,0.81	1.18	0.68,2.04	0.39	0.22,0.71	0.98	0.53,1.8
Race & Ethnicity												
NH White	Ref.		Ref.		Ref.		Ref.		Ref.		Ref.	
NH Black	1.18	1.04,1.34	1.01	0.88,1.16	0.98	0.82,1.17	0.69	0.56,0.83	1.06	0.88,1.28	0.76	0.62,0.94
Hispanic	0.42	0.33,0.53	0.45	0.35,0.59	0.14	0.08,0.24	0.15	0.09,0.25	0.15	0.09,0.26	0.17	0.09,0.3
Other/multiple races	0.51	0.4,0.65	0.75	0.58,0.96	0.33	0.22,0.5	0.52	0.34,0.79	0.37	0.24,0.55	0.61	0.4,0.95
Median income^1^												
1st quartile	Ref.		Ref.		Ref.		Ref.		Ref.		Ref.	
2nd quartile	0.61	0.53,0.7	0.87	0.75,1.01	0.46	0.37,0.57	0.76	0.61,0.95	0.47	0.38,0.58	0.79	0.63,1
3rd quartile	0.45	0.38,0.53	0.76	0.64,0.9	0.37	0.29,0.47	0.82	0.64,1.05	0.33	0.26,0.43	0.76	0.58,0.99
4th quartile	0.41	0.34,0.49	0.73	0.6,0.9	0.24	0.17,0.33	0.61	0.44,0.86	0.21	0.15,0.29	0.56	0.39,0.81
Primary insurance												
Private	Ref.		Ref.		Ref.		Ref.		Ref.		Ref.	
Medicaid	2.82	2.48,3.21	2.16	1.85,2.53	7.94	6.2,10.17	5.25	3.91,7.06	8.88	6.77,11.65	5.76	4.2,7.89
Self-pay/none	1.59	1.16,2.18	1.61	1.17,2.2	2.47	1.42,4.3	2.36	1.35,4.12	2.40	1.29,4.47	2.35	1.26,4.37
Urban residence	1.04	0.7,1.53	1.25	0.84,1.85	1.04	0.58,1.88	1.64	0.9,2.99	1.02	0.55,1.89	1.50	0.81,2.8
Comorbidities during delivery												
MH condition	8.78	7.81,9.86	6.58	5.76,7.53	7.14	6.01,8.48	3.07	2.5,3.77	11.23	9.39,13.43	5.42	0.59,6.71
SUD	6.14	5.29,7.13	2.07	1.74,2.45	23.54	19.89,27.87	7.39	5.99,9.13	20.29	16.95,24.29	5.30	4.27,6.59
Stillbirth	3.49	2.41,5.06	2.24	1.48,3.4	3.32	1.83,5.99	1.86	1,3.46	2.52	1.3,4.87	1.42	0.7,2.88

Note: SMM,severe maternal morbidity; NH,non-Hispanic; MH,mental health; SUD,substance use disorder.

1 Income is household median for zip code. Adjusted models include all variables in the table. Standard errors adjusted for individual-level clustering. Unadjusted models show the odds of SMM for each characteristic.

Source: Healthcare Cost and Utilization Project (HCUP),Maryland Inpatient Database,2016–2019. N = 197,749 deliveries in 2016–2018.

## Data Availability

The data that support the findings of this study are publicly available from The Agency for Healthcare Research and Quality, Healthcare Cost and Utilization Project. HCUP State Inpatient Databases (SID). Healthcare Cost and Utilization Project (HCUP). 2015–2019. Agency for Healthcare Research and Quality, Rockville, MD. www.hcup-us.ahrq.gov/sidoverview.jsp

## Abbreviations

SMM: Severe Maternal Morbidity
